# Is parental competitive ability in winter negatively affected by previous springs’ family size?

**DOI:** 10.1002/ece3.2752

**Published:** 2017-02-03

**Authors:** Rienk W. Fokkema, Richard Ubels, Joost M. Tinbergen

**Affiliations:** ^1^ Conservation Ecology GroupGroningen Institute for Evolutionary Life SciencesUniversity of GroningenGroningenThe Netherlands

**Keywords:** brood size manipulation, density dependence, frequency dependence, parental care, social environment

## Abstract

Reproductive behavior cannot be understood without taking the local level of competition into account. Experimental work in great tits (*Parus major*) showed that (1) a survival cost of reproduction was paid in environments with high levels of competition during the winter period and (2) experimentally manipulated family size negatively affected the ability of parents to compete for preferred breeding boxes in the next spring. The fact that survival was affected in winter suggests that the competitive ability of parents in winter may also be affected by previous reproductive effort. In this study, we aim to investigate whether (1) such carryover effects of family size on the ability of parents to compete for resources in the winter period occurred and (2) this could explain the occurrence of a survival cost of reproduction under increased competition. During two study years, we manipulated the size of in total 168 great tit broods. Next, in winter, we induced competition among the parents by drastically reducing the availability of roosting boxes in their local environment for one week. Contrary to our expectation, we found no negative effect of family size manipulation on the probability of parents to obtain a roosting box. In line with previous work, we did find that a survival cost of reproduction was paid only in plots in which competition for roosting boxes was shortly increased. Our findings thus add to the scarce experimental evidence that survival cost of reproduction are paid under higher levels of local competition but this could not be linked to a reduced competitive ability of parents in winter.

## 1 Introduction

Competition within the social environment of a parent may be an important selective force on its reproductive behavior (Nicolaus et al., 2012; Svensson & Sheldon, 1998; Wilson, 2014). Life history theory (Roff, 1992; Schaffer & Rosenzweig, 1977; Stearns, 1992) identifies two fundamental trade‐offs that determine individual reproductive decisions: (1) the trade‐off between quality and quantity of offspring (Lack, 1947) and (2) the trade‐off between current and future reproduction (Williams, 1966). Here, we focus on the mechanism behind the parental cost of reproduction, an important component of the second trade‐off.

The trade‐off between current and future reproduction implies that if a parent increases its investment into current reproduction, this leads to physiological costs for the parent; in turn, these physiological costs are expected to lead to fitness costs of reproduction (i.e., survival or fecundity costs; Williams, 1966; for review, see Speakman, 2008). The actual empirical evidence, especially for a survival cost of reproduction, has proved mixed; however (avian: Linden & Moller, 1989; Dijkstra et al., 1990; Stearns, 1992; Golet, Irons, & Estes, 1998; Parejo & Danchin, 2006; Santos & Nakagawa, 2012; mammals: Stearns, 1992; Hamel et al., 2010), in some populations, a survival cost of reproduction has been detected, but not in others.

One reason for this discrepancy may be that a survival cost of reproduction may only occur when competition in the social environment is high (Nicolaus et al., 2012; Oksanen, Koivula, Koskela, & Mappes, 2007). In experimental work by Nicolaus et al. (2012) on great tits (*Parus major*), it was shown that parents, during the winter period, paid a survival cost of reproduction in areas with increased competition, but not in areas with low competition. The authors hypothesized that family size negatively affected the competitive ability of parents, and under high competition, this led to a survival cost of reproduction. If so, such carryover effects (Harrison, Blount, Inger, Norris, & Bearhop, 2011; O'Connor, Norris, Crossin, & Cooke, 2014) could provide a causal explanation for the general pattern that parents reduce their reproductive investment at higher population density and presumably competition (e.g., avian: Kluijver, 1951; Perrins, 1965; Both, Tinbergen, & Visser, 2000; Nicolaus, Brommer, Ubels, Tinbergen, & Dingemanse, 2013; mammals: Morris, 1989; Koskela, Mappes, & Ylönen, 1999; Bonenfant et al., 2009).

In a recent study, we found first evidence that indeed family size negatively affects the competitive ability of parents in the next spring (Fokkema, Ubels, & Tinbergen, 2016). Consistently, over two study years, experimentally manipulated family size had a negative effect on the ability of great tit (*Parus major*) parents to claim a high‐quality breeding box the following spring. The result of Nicolaus et al. (2012) that a survival cost of reproduction was paid only in environments with high competition in winter suggests that similar negative effects of family size on the ability of parents to compete for resources in winter may exist. Here, we aim to directly test whether (1) such carryover effects of family size on the ability of parents to compete for resources in the winter period occurred and (2) this could explain the occurrence of a survival cost of reproduction under increased competition in winter (as observed by Nicolaus et al., 2012). By doing this, we gain insight when in the life cycle of a parent competitive ability is affected by earlier reproductive effort. This knowledge is vital to predict selection on reproductive investment under local competition.

One important resource for which competition in winter may occur is the availability of roosting boxes. Roosting in a nest box, as opposed to roosting outside, may enhance winter survival of birds by decreasing thermoregulatory costs and the risk of predation (Atema, Van Noordwijk, Boonekamp, & Verhulst, 2016; Drent, 1987; Mainwaring, 2011). We expected that if the availability of roosting boxes was limited, experimentally manipulated family size would negatively affect the ability of parents to claim a roosting box in the subsequent winter. This in turn could result in a survival cost of reproduction for the parents involved.

To test our expectation, we experimentally manipulated family size during two study years. Subsequently, at midwinter, we induced competition among the manipulated great tit parents for roosting boxes for a short period by strongly reducing the availability of roosting boxes in half of the study area. We next quantified the effect of family size manipulation on the ability of parents to claim a roosting box and on the apparent survival of parents in relation to the increased competition for roosting boxes.

## 2 Methods

### 2.1 Study area and study population

We studied a nest box breeding great tit population in the Lauwersmeer area in the north of the Netherlands (coordinates: 53°23′N, 6°14′E). The area of approximately 24 km^2^ was planted in 1969 resulting in a relatively young (approx. 40 years old) mainly deciduous forest interspersed with grassy areas. The nest box population comprised 12 nonadjacent plots with 50 boxes each, resulting in 600 nest boxes in total (Nicolaus et al., 2009).

Ethical permission for this study was given by the Animal Experiments Committee (DEC project: 5548F).

### 2.2 Breeding season

#### 2.2.1 Monitoring egg laying and breeding

We checked all 600 nest boxes every week during the breeding season of 2012, 2013, and 2014. If eggs were encountered in a nest box, we calculated the first egg laying date, assuming that one egg was laid a day. Next, as soon as we detected that clutches were incubated, we calculated an expected hatching date. This calculation was made on the basis of the first egg laying date and the clutch size, assuming that breeding started directly after the last egg was laid, and that the incubation period lasted 12 days (e.g., de Heij, van den Hout, & Tinbergen, 2006). We checked all incubated nests daily 1–2 days before the expected hatching date (day 0) and this continued until the first egg hatched.

#### 2.2.2 Family size manipulation

Five days after hatching, we visited the nest again, recorded the number of nestlings, and weighed the entire brood (mass ±0.1 g). Using these data (see below), we manipulated the family sizes the next day (day 6). Family sizes were manipulated as follows. When the nestlings were 6 days old, we matched a set of three nests with a similar hatching date according to the number of nestlings, clutch size, and brood weight (hereafter called “trio”; for analysis purposes, each trio was assigned a number to correct for nonindependence, see “2.5.2”). Within the trio, we randomly assigned nest treatment and the nestlings to exchange: one family was enlarged, one family was reduced, and one family was kept as a control. We exchanged three nestlings in most trios (2012: *N* = 28 trios, 2013: *N* = 21 trios; both years: average number of nestlings pre‐exchange: Reduced: 8.5, Control: 8.3, Enlarged: 8.4; postexchange: *R*: 5.5, *C*: 8.3, *E*: 11.4). In some cases, however, we exchanged two nestlings (2012: *N* = 3 trios, 2013: *N* = 4 trios; both years: average number of nestlings pre‐exchange: *R*: 6.4, *C*: 6.6, *E*: 6.6; postexchange: *R*: 4.4, *C*: 6.6, *E*: 8.6). We did this to prevent brood desertion when, after reduction, the family size would be less than five nestlings (Verboven & Tinbergen, 2002). To ensure that broods were disturbed to a similar extent and that the fraction of own nestlings relative to the total number of offspring remained approximately the same, we also exchanged four nestlings of the control brood, two with two nestlings of the reduced brood and two with two nestlings of the enlarged brood (for further details, see de Jong, Fokkema, Ubels, van der Velde, & Tinbergen, 2014; Fokkema et al., 2016).

We subsequently measured whether the family size manipulation (hereafter termed “FS manipulation”) successfully increased parental feeding effort judged by three components, the number of visits made by each parent per day, the gain in weight of the brood after FS manipulation, and the number of fledglings produced. The number of visits made by each parent and the number of fledglings produced successfully increased with FS manipulation, and no such effect was found on the gain in weight of the manipulated broods (see Appendix S1).

#### 2.2.3 Identification of parents and providing RFID transponder rings

**Figure 1:**
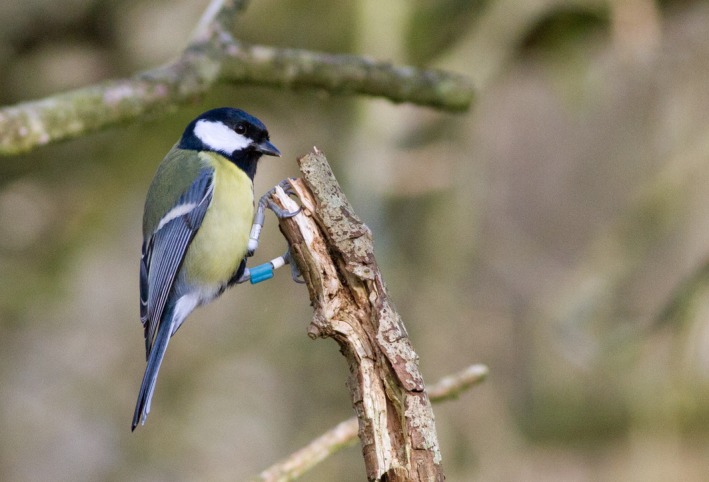
A great tit (*Parus major*) with an RFID transponder ring (light blue). The transponder ring enabled measurements of the number of feeding visits made by parents in response to family size manipulation and identification without disturbance when roosting. On the legs of the bird, additionally, a color ring and an aluminum ring were fitted to enable visual identification. Picture taken by: Richard Ubels

During the nest box checks, we visually identified the incubating female, if possible, when she was sitting tight on the eggs, on the basis of a previously applied unique combination of color rings to her legs. The day after FS manipulation (nestlings 7 days old), we additionally caught both parents (also the previously identified females during incubation) using spring traps inside the nest box. If parents could not be caught the day after FS manipulation, a second attempt was made 2 days later. When caught, parents were identified based on the existing identification rings (aluminum ring with unique inscription and three plastic color rings). If not yet ringed, we provided parents with identification rings. In 2013, we altered the color ring scheme and 146 caught parents which raised a manipulated brood were provided with an RFID transponder ring (type: EM4102 bird PIT tag 2.6 mm, manufactured by IB technology, Eccel Technology Limited; each bird was provided with a transponder ring, an aluminum ring, and two plastic color rings; see Figure 1). These transponder rings enabled us to measure the effects of FS manipulation on parental feeding effort during the breeding season (see: Appendix S1) and identification while roosting in winter (see “2.3.2”). Some parents could not be caught at all, and these were identified if possible, using binoculars (again based on existing color rings).

#### 2.2.4 Injuries due to transponders

Unexpectedly, 9% of the parents with a transponder ring in 2013 developed injuries after the breeding season (swollen leg, sometimes necrosis; injuries first detected in November) on the leg to which this ring was fitted (13 of the 146 parents with a transponder ring). We showed that the feeding effort of parents in 2013 did increase with FS manipulation (Appendix S1) and that the manipulation groups did not differ in their probability to get injured (χ^2^_*df*NA_ = 2.92, *p* = .25, degrees of freedom could not be calculated, see “2.5.3”). Any effects of injuries due to the transponders on the competitive ability of parents or their local survival probability were thus not likely to differ between the FS manipulation groups. Injured birds were treated by removing their transponder ring. We additionally removed the plastic color ring attached to the same leg as the transponder for birds with no injuries. The latter treatment did not prevent injuries altogether. In 2014, for a different experiment, two parents developed injuries (of the 184 parents provided with a single transponder ring this year). In general, the injuries did not seem to cause increased mortality and four of the five parents with serious injuries (necrotic legs) were even able to start a new brood the following season (overall, 10 of the 13 parents observed with injuries were able to start a brood in 2014 (77%), average local survival of parents in this period for 2010–2012: (43%)).

### 2.3 Midwinter competition experiment

#### 2.3.1 Inducing competition for roosting boxes

**Figure 2:**
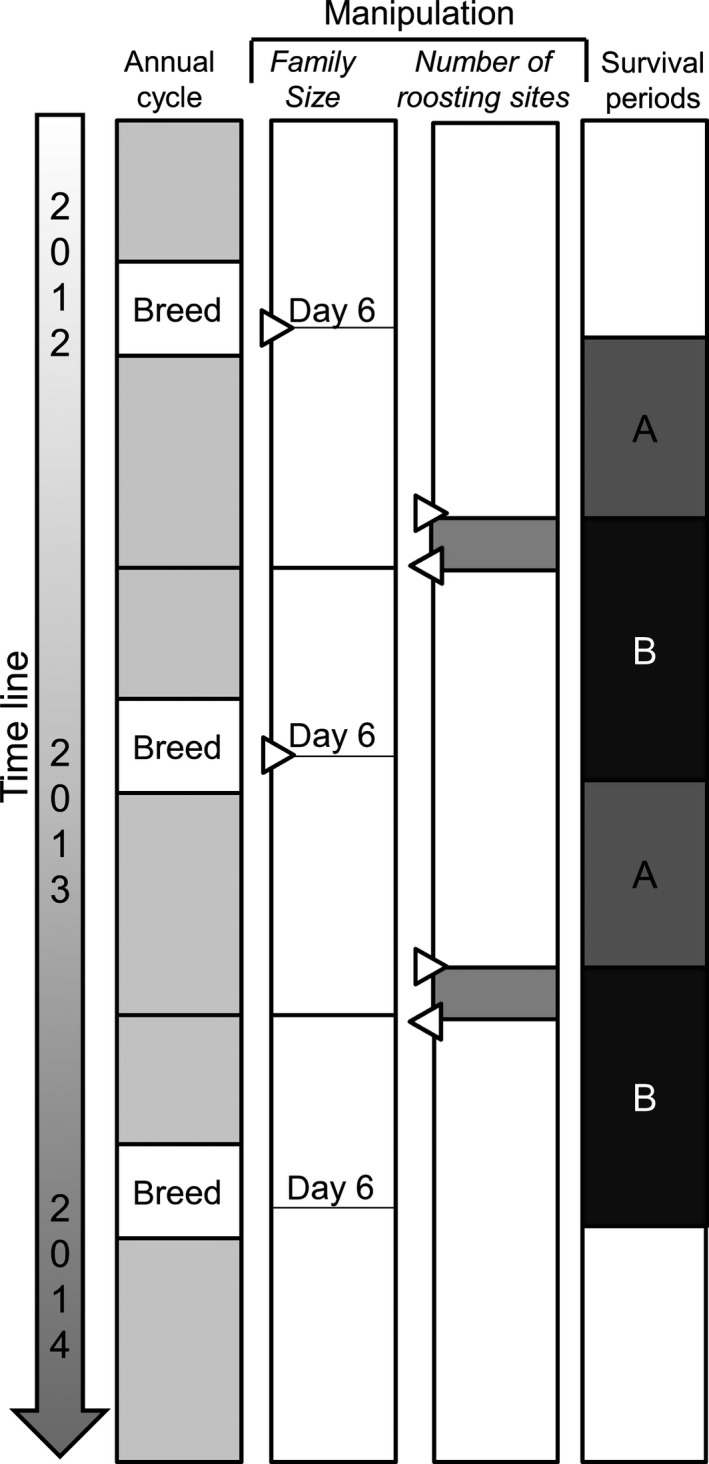
Time line of the experiments relative to the annual cycle. In 2012 and 2013, family size was manipulated when the nestlings were 6 days old (second column, black lines with triangles pointing right). In the subsequent winter, the number of roosting boxes was reduced by 80% in half of the study area (“the experimental plots”; third column, right‐pointing triangles). The other half of the plots were kept as a control. One week later, we restored the number of roosting boxes (left‐pointing triangles). We measured the local survival probability of parents during two periods (fourth column): (A) from the breeding season until the time point competition for roosting boxes was induced and B) from the time point that competition was induced until the following breeding season. For further explanation, see 2

At the beginning of December in 2012 and 2013 (termed “midwinter” hereafter), we spend two consecutive evenings checking all nest boxes in our study area for roosting birds (12 study plots; 50 boxes per plot). Two nights after this roost check, we induced competition in six of the 12 study plots in the area (termed “experimental plots” hereafter; the uneven numbered plots in 2012 and the even numbered plots in 2013) and kept the remaining six plots as controls (Figure 2).

We induced competition in the experimental plots by, at daytime, plugging up the entrance hole of all 50 boxes available per study plot and redistributing 10 new roosting boxes at new locations in the plot. We chose to reduce the number of boxes from 50 to 10 to induce competition in all study plots as the number of sleeping great tits per plot differed markedly (between eight and 44 great tits roosted per study plot in earlier roost checks at midwinter in 2010 and 2011). We chose the locations of the new boxes relative to a randomly assigned selection of 10 of the old nest box locations in the plot. We put up the new boxes 25 m to the northeast of these locations. If this location was not suitable (e.g., no trees available), we placed the new box 25 m to the southeast. Analysis showed that the FS manipulation groups did not differ in the distance from the box in which parents roosted before the experiment to the nearest available new box (linear model: *F*_(2,63)_ = 0.35, *p* = .70, average distance to a new roosting box: 58 m).

During the week of competition, the average local temperatures were the following in 2012: *T*_min_ = 0.9**°**C, *T*_max_ = 4.9**°**C and 2013: *T*_min_ = 3.4**°**C, *T*_max_ = 7.5**°**C. In 2013 but not in 2012, throughout the study area, we provided supplementary food at feeding stations for a different experiment. Using transponder readers, we could identify which manipulated parents visited these feeding stations and how often. Based on this data, we found no evidence that parents of the manipulation groups used the supplementary food differentially (generalized linear model with quasi‐Poisson error structure: *F*_(2,23)_ = 0.02, *p* = .98, average number of visits to feeder: 237). These differences in winter conditions between study years were taken into account in our analyses by including study year and the interaction between family size manipulation and study year (see “Statistics”). Few natural cavities were likely available to the birds as alternative to the nest boxes as the forest is relatively young ((Newton, 1994); see “Study area and study population”).

#### 2.3.2 Occupation rate of the roosting boxes after competition was induced

In 2013, spread over the week that competition for roosting boxes was induced, we performed two roosting checks in the experimental plots to monitor box occupation over time (1–2 and 3–4 days after competition was induced). We used handheld readers (type: LID575‐ISO; manufactured by Dorset Identification b.v.) which could read the code emitted by the transponder of the parents through the bottom of the box (to minimize the disturbance of the roosting birds). Our data show that parents found the new boxes soon after competition was induced (1–2 nights after roosting 70% of the manipulated parents (*N* = 10) observed in the final night check at the end of the experiment (see section below) were detected). We never observed that roost boxes had different owners during the experiment. Those parents that were detected multiple times in the roost checks during the experiment were observed to roost in the same box.

#### 2.3.3 Determining the winners of the competition for roosting boxes

In both years, seven nights after competition for roosting boxes was induced, we performed a final roost check in the experimental and the control plots to determine which manipulated parents were able to claim a roosting box (the winners). During this roost check, like in the roost check before the onset of the midwinter competition experiment, boxes were opened and birds were taken out and identified (both years combined: *N* = 43 parents observed roosting in the control plots of 50 parents observed before competition was induced; *N* = 27 parents observed roosting in the experimental plots of 66 parents observed before). None of the manipulated parents moved between plots during the experiment.

### 2.4 Parental fitness components

To assess whether a survival cost of reproduction was paid before or after the time point that competition for roosting boxes was experimentally induced, we calculated (1) the local survival probability of both parents over the period from the breeding season (*N* = 323) until midwinter (*N* = 155) and (2) the local survival probability of both parents over the period from midwinter until the following breeding season (*N* = 46; Figure 2). For our measure of local survival of parents from the breeding season until midwinter, we deemed all parents observed roosting in the first night check as alive plus those later seen alive during the breeding season. Local survival after midwinter until the following breeding season was determined based on recaptures during the breeding season (local survival in our study thus corresponds to apparent survival). Mark–recapture models were not used to estimate parental local survival because the adult detection probability if alive in the breeding season is high in this population (0.897, SE = 0.055 see Tinbergen & Sanz, 2004).

### 2.5 Statistics

We used R (version 3.2.3; R Core Team 2015) and the package “lme4” (Bates, Mächler, Bolker, & Walker, 2015) to create the mixed models. The effects of FS manipulation on the probability of parents to claim a roosting box and on parental local survival were analyzed using generalized linear mixed effects models (GLMER) with a binomial error structure.

#### 2.5.1 Predictor variables included

Within all analyses, FS manipulation was included as a continuous variable because of our directional expectation (directional statistical tests, see Fokkema et al., 2016; Knowles, Nakagawa, & Sheldon, 2009), and we allowed for nonlinear effects by including a quadratic effect of FS manipulation. Next to this, we included three other predictor variables. We included the factors, study year and sex of the parent, in all analyses and the factor plot treatment (experimental or control plots) in the analyses of the probability of parents to claim a roosting box and parental local survival after midwinter. All three above described factors were tested as main effect and in interaction with FS manipulation and FS manipulation^2^.

#### 2.5.2 Random effects included

We included three random variables in all analyses: (1) “trio” number, this factor was included to correct for nonindependence of the matched trios of nests (see 2 “family size manipulation”), (2) brood ID, this identification number for the brood raised was included to account for the fact that some parents had a shared history, and (3) individual ID, this identification number for the individual parent was included as a factor to account for the fact that some individuals were observed during both experimental years (*N* = 21 individuals with repeated measurements within the analysis of local survival until the midwinter and *N* = 5 individuals with repeated measurements in both the analysis of the probability of a parent to claim a roosting box and the probability of a parent to survive after midwinter).

#### 2.5.3 Model selection

Our aim was to get the most accurate estimate of the effects of FS manipulation. We therefore tested which predictor variables, that did not significantly aid in estimating the effect of FS manipulation, could be eliminated. We first tested whether the interactions between FS manipulation^2^ and all included predictor variables could be eliminated in order of significance. Next, we eliminated the interactions between FS manipulation and the predictor variables if nonsignificant. Then, FS manipulation^2^ was removed if possible and finally all other predictor variables were removed in order of their significance. This backward elimination procedure was carried out on the basis of likelihood ratio tests. We kept the random effects in the models at all times during model selection as these were there to correct for nonindependence in the dataset (as in Fokkema et al., 2016).

**Figure 3:**
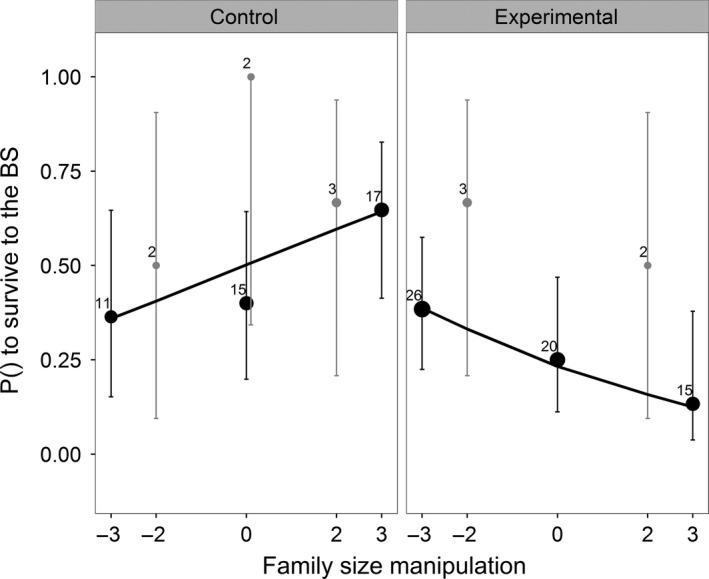
The effect of family size manipulation on the local survival probability of parents from midwinter until the next breeding season. A survival cost of reproduction was only paid in the plots in which competition was induced. Black dots depict manipulations in which three nestlings were exchanged; gray dots depict manipulations in which two nestlings were exchanged. Sample size is indicated by symbol size and the numbers next to the 95% confidence intervals. The solid line depicts the predicted response calculated on the basis of the final selected model

The chi‐square goodness of fit test presented in the 2 section “injuries due to transponders” was carried out using simulated *p*‐values due to the low sample size. When using this method, the degrees of freedom cannot be given and are presented as NA (Hope, 1968). Package “ggplot2” (Wickham, 2009) was used to construct the figures. We calculated the solid lines in Figure 3 using the predict function of package “lme4” on the basis of the selected models.

## 3 Results

### 3.1 Family size manipulation and the local survival probability of parents until midwinter

We found no effects of FS manipulation on the local survival probability of parents until midwinter (FS manipulation: χ^2^_*df*1_ = 0.53, *p* = .47; FS manipulation^2^: χ^2^_*df*1_ = 0.001, *p* = .97; average local survival probability of parents: reduced: 0.51 (95% CI = 0.42, 0.61), control: 0.47 (95% CI = 0.37, 0.56), enlarged: 0.46 (95% CI = 0.37, 0.55), for the latter comparison, we grouped the −3/−2 and the +2/+3 FS manipulations). We further found no indication that the direction of the effect of FS manipulation on parental local survival differed depending on the study year or sex of the parent or that the factors, sex of the parent and study year, independently played a role.

### 3.2 Midwinter experiment: competition for roosting boxes

#### 3.2.1 Family size manipulation and the probability to occupy a roosting box before competition for roosting boxes was induced

Controlling for effects of study year and sex of the parent (lower probability of roosting boxes to be occupied in 2013: intercept: −0.91 ± 0.25, β = −0.60 ± 0.31, χ^2^_*df*1_= 4.02, *p* < .05; higher probability of males to occupy a roosting box: β = 0.95 ± 0.27, χ^2^_*df*1_ = 13.58, *p* < .001), we found no effect of FS manipulation on the probability to encounter a parent in a roosting box in the first roost check (FS manipulation: χ^2^_*df*1_ = 0.87, *p* = .35; FS manipulation^2^: χ^2^_*df*1_ = 0.003, *p* = .96; average probability of parents to occupy a roosting box: reduced: 0.39 (95% CI = 0.31, 0.49), control: 0.35 (95% CI = 0.27, 0.45), enlarged: 0.33 (95% CI = 0.25, 0.43), for the latter comparison, we grouped the −3/−2 and the +2/+3 FS manipulations). We further found no indication that the direction of the effect of FS manipulation on the probability to observe parents in a roosting box differed depending on the study year or sex of the parent.

#### 3.2.2 Fraction of roosting boxes occupied after competition for roosting boxes was induced

Consistent with what we would expect if competition occurred, the fraction of roosting boxes occupied by great tits in the experimental plots significantly increased (see Table 1 for absolute numbers of roosting boxes occupied; chi‐square goodness of fit test: 2012: χ^2^_*df*1_ **= **5.71, *p* < .05; 2013: χ^2^_*df*1_ **= **23.77, *p* < .001). In 2012, in the control plots, the fraction of occupied roosting boxes by great tits decreased slightly (χ^2^_*df*1_ **= **6.18, *p* < .05), while in 2013, it remained constant (χ^2^_*df*1_ **= **1.33, *p* **= **.25).

**Table 1:** Overview of the number of roosting boxes occupied in both the control and experimental plots by great and blue tits before and after competition for roosting boxes was induced in the experimental plots

Control plots	Before competition (300 boxes available)			After competition (300 boxes available)		
Year	Great tit	Blue tit	Empty	Great tit	Blue tit	Empty
2012	183	39	78	162	42	96
2013	145	33	122	135	27	138

Experimental plots	Before competition (300 boxes available)			After competition (60 boxes available)		
Year	Great tit	Blue tit	Empty	Great tit	Blue tit	Empty
2012	213	31	56	51	4	5
2013	136	41	123	46	7	7

The fraction of roosting boxes occupied by the subdominant blue tit (*Cyanistes caeruleus,* the only species that makes use of roosting boxes in our study area besides the great tit) stayed constant in the experimental plots (2012: χ^2^_*df*1_ = 0.29, *p* = .59; 2013: χ^2^_*df*1_ = 0.20, *p* = .65) and in the control plots (2012: χ^2^_*df*1_ = 0.27, *p* = .61; 2013: χ^2^_*df*_  = 1.23, *p* = .27). The number of empty boxes in the experimental plots decreased significantly (2012: χ^2^_*df*1_ = 4.21, *p* < .05; 2013: χ^2^_*df*1_ = 21.34, *p* < .001), while in the control plots, the number of empty boxes increased in 2012 (χ^2^_*df*1_ = 5.61, *p* < .05) and stayed constant in 2013 (χ^2^_*df*1_ = 3.54, *p* = .06).

#### 3.2.3 Family size manipulation and the probability of parents to claim a scarce roosting box

Against expectation, after competition for roosting boxes was induced, we found no effect of FS manipulation on the ability of parents to claim a roosting box (corrected for effects of sex and plot treatment: Table 2; average probability to obtain a box: experimental plots: R: 0.38 (95% CI** = **0.23, 0.56), C: 0.45 (95% CI** = **0.26, 0.66), E: 0.47 (95% CI** = **0.26, 0.69); control plots: R: 0.69 (95% CI** = **0.42, 0.87), C: 0.88 (95% CI** = **0.66, 0.97), E: 0.90 (95% CI** = **0.70, 0.97)). We further found no evidence that the direction of the effect of FS manipulation on the probability of parents to claim a roosting box differed with plot treatment, year, or sex.

**Table 2:** Outcome of the generalized linear mixed effects model describing the effects of family size manipulation on the probability of parents to claim a roosting box (*N* = 116 parents)

Variable	Estimate (β ± SE)	Δχ ^2^	*df*	*p*
Intercept	0.90 (0.47)			
Family size manipulation	0.14 (0.10)	2.15	1	.14
Sex		10.89	1	<.001
Male effect (relative to female)	1.55 (0.50)			
Plot treatment		23.64	1	<.001
Experimental plots (relative to control plots)	−2.16 (0.57)			

The probability to claim a roosting box was much lower in the experimental plots, but no effect of family size manipulation could be detected. The variance of the random effect trio was 8.3e^−2^, the variance of the random effect brood id was 0, and the variance of the random effect individual id was 2.3e^−9^.

Rejected terms: manipulation^2^ × plot treatment (*df*1), manipulation^2^ × sex (*df* 1), manipulation^2^ × year (*df*1), manipulation × plot treatment (*df*1), manipulation × sex (*df*1), manipulation × year (*df*1), manipulation^2^ (*df*1), manipulation (*df*1), year (*df*1).

### 3.3 Effects of family size manipulation on fitness components after midwinter

**Figure 4:**
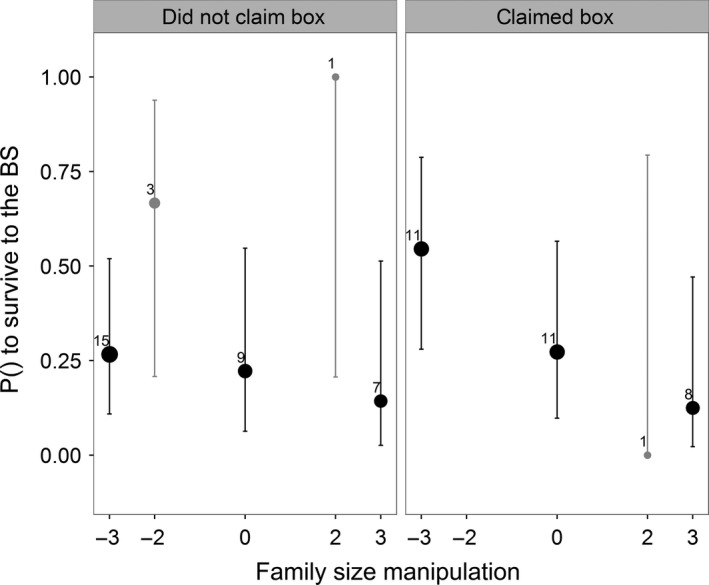
The effect of family size manipulation on the local survival probability of parents that *did not* and parents that *did* occupy a roosting box after competition for them was induced. The data suggest a survival cost of reproduction for those birds that did occupy a roosting box in contrast to those that did not occupy a roosting box. Black dots depict manipulations in which three nestlings were exchanged; gray dots depict manipulations in which two nestlings were exchanged. Sample size is indicated by symbol size and the numbers next to the 95% confidence intervals. The solid line depicts the predicted response calculated on the basis of the final selected model

Controlled for effects of study year, we found that experimentally manipulated family size did have a consistent negative effect on the local survival probability of parents from midwinter until the breeding season in plots where we induced competition for roosting boxes, but not on the local survival probability of parents in control plots (Figure 3; Table 3). In the control plots, effects of FS manipulation seemed to work in the opposite direction. The effect of FS manipulation did not differ between study years or between the sexes. There further was no evidence for a nonlinear effect of FS manipulation, nor for a difference between the sexes. Further analysis showed a trend that the survival cost of reproduction in the experimental plots was paid only within the group of parents that were able to claim a roosting box during the experiment (Figure 4; controlled for year effect; FS manipulation × claimed box: intercept: −7.18 ± 4.39, β = −1.45 ± 1.53, χ^2^_*df*1_ = 2.82, *p* = .09).

**Table 3:** Outcome of the generalized linear mixed effects model describing the effects of family size manipulation on the local survival probability of parents resident in the control and the experimental plots from midwinter to the following breeding season (*N* = 116 parents)

Variable	Estimate (β ± SE)	Δχ^2^	*df*	*p*
Intercept	−1.16 (0.55)			
Family size manipulation	0.27 (0.18)			
Family size manipulation × plot treatment		4.80	1	<.05
Experimental plots (relative to control plots)	−0.55 (0.26)			
Year		25.15	1	<.001
2013 (relative to 2012)	3.34 (0.83)			
Plot treatment				
Experimental (relative to control)	−1.73 (0.66)			

The variance explained by the random effect trio was 1.18, the variance of the random effect brood id was 0, and the variance of the random effect ring number which coded for the individual was 7.67e^−15^.

Rejected terms: manipulation^2^ × sex (*df*1), manipulation^2^ × year (*df*1), manipulation^2^ × plot treatment (*df*1), manipulation × sex (*df*1), manipulation × year (*df*1), manipulation^2^ (*df*1), sex (*df*1).

There was no indication that the observed effects in the experimental plots of FS size manipulation on parental local survival from midwinter until the following breeding season were the consequence of selective dispersal rather than selective mortality. In both the control and the experimental plots, there was no effect of FS manipulation on the distance moved between the roosting box in which a parent was observed at midwinter and the box it used for breeding the following spring (linear model: FS manipulation × plot treatment: *F*_(1,41)_ = 0.31, *p* = .58, FS manipulation: *F*_(1,42)_ = 1.28, *p* = .26; controlled for effects of sex and year; average distance moved to breeding box: 84 m).

## 4 Discussion

The aim of this study was to test whether (1) family size affects the ability of great tit parents to compete for roosting sites in the winter period and (2) whether this could explain the occurrence of a survival cost of reproduction under increased competition in winter (as shown by Nicolaus et al., 2012). Such a carryover effect of family size on parental competitive ability and subsequent fitness (Harrison et al., 2011; O'Connor et al., 2014) could provide a causal explanation why selection would favor smaller families at higher population density (e.g., avian: Kluijver, 1951; Perrins, 1965; Both et al., 2000; Nicolaus et al., 2013; mammals: Morris, 1989; Koskela et al., 1999; Bonenfant et al., 2009). After experimental reduction of the number of roosting boxes in winter, we found that (1) prior family size manipulation did not affect the ability of great tit parents to claim one of the scarce roosting boxes, but (2) we did find that the short period of increased competition for roosting boxes resulted in a survival cost of reproduction, strengthening the scarce experimental evidence (Nicolaus et al., 2012) that survival cost of reproduction depend on the competitive situation. Yet, the question how a survival cost of reproduction is paid under competition remains unsolved; it was not due to a reduced ability of parents to claim a scarce roosting box.

### 4.1 Family size and competitive ability

Here, we explore two possible reasons why a negative effect of family size on the ability of parents to claim a roosting box was not apparent.

#### 4.1.1 Were the costs of reproduction already paid before midwinter?

One potential reason for not finding a negative effect of family size on the competitive ability of parents is that parents already paid a survival and/or fecundity cost of reproduction in full before midwinter. This could happen because parents with different reproductive efforts experienced differential survival till midwinter, but we found no evidence that this was the case. Alternatively, it could happen because parents as a consequence of the manipulation of the size of their first brood differentially invested in late broods within the same breeding season. This was the case in 2013 (see Appendix S1) but not in 2012. In 2013, parents in the different manipulation groups may thus have had a similar reproductive investment over the whole breeding season. Despite this, we found no difference between the years 2012 and 2013 in the negative effect of family size manipulation on the local survival of parents *after* midwinter in the plots in which competition was induced. In line with previous studies (Fokkema et al., 2016; Nicolaus et al., 2012), parents did thus not fully compensate for effects of family size manipulation in 2013 by foregoing a late brood within the same season. We thus have no evidence that costs of reproduction were already paid in full before midwinter.

#### 4.1.2 Was there competition among parents for roosting boxes?

It may be that effects of family size on parental competitive ability were there but that we could not detect them, because we did not successfully induce competition among the parents by reducing the availability of roosting boxes. It is well established that roosting boxes are an important resource for great tits in winter to evade predation and reduce thermoregulatory costs (Drent, 1987; Mainwaring, 2011). Potentially, roosting boxes were less important in this respect in 2013 than in 2012, because in this year, the local temperature in winter was higher and supplementary food was provided (for a different study). Consistent with this, during the first night check in 2013 at midwinter, the occupancy rate of roosting boxes was lower than in 2012. However, we found no year difference in the effect of family size manipulation on the probability that parents claimed a roosting box, and also not in their local survival probability from midwinter to the following breeding season. This indicates that the difference in winter conditions between study years did not affect the outcome of our experiment.

Our results indicate that we were successful in inducing competition among the group of great tits that did roost in boxes because (1) the newly available roosting boxes were immediately found and occupied (data of birds provided with a transponder in 2013), (2) the fraction of boxes occupied by great tits increased in response to the reduction in the number of roosting boxes in both years, and (3) males (the dominant sex) were more successful than females in securing a scarce roosting box in both study years. Unexpectedly in both years, with increased competition, some supposedly subdominant blue tits (see Kempenaers & Dhondt, 1991) were still able to claim a roosting box and a small number of the available roosting boxes remained empty (Table 1). Overall, we conclude that, in both study years, we were successful in inducing competition among the manipulated great tit parents by reducing the amount of available roosting boxes.

### 4.2 Survival cost of reproduction under competition

Although we found no clear effect of family size on the ability of parents to claim a roosting box, we did find that parents that managed to claim a roosting box in plots in which we induced competition paid a survival cost of reproduction, whereas this effect was absent in the control plots.

#### 4.2.1 Dispersal or mortality?

Important to address first is whether the observed negative effect of family size manipulation on the local survival of parents in the plots in which competition was induced was due to increased mortality or to dispersal. Great tits parents are known to have a very limited breeding dispersal (own study population: Tinbergen & Sanz, 2004; Andreu & Barba, 2006), but it could be that in response to the sudden drop in the number of available roosting sites, parents moved elsewhere. We could not detect any movements of parents, however, between study plots in which competition for roosting boxes was induced and control plots; even though in the control plots, empty boxes were potentially available (Table 1). We further found no evidence for plot treatment‐specific effects of family size manipulation on the dispersal distance of parents between the box in which they roosted at midwinter and the box they used for breeding the next spring. This indicates that mortality effects and not dispersal effects explain the observed difference in effect of family size manipulation on the local survival rate of parents after midwinter between the experimental and control plots.

#### 4.2.2 Experimental evidence for a survival cost of reproduction under competition

Our results are in line with the results of the experimental study by Nicolaus et al. (2012) in the same great tit population. In their study, competition was manipulated by experimentally altering local sex ratios as such that male‐biased, control, and female‐biased study plots were created. Nicolaus et al. (2012) found that a survival cost of reproduction was only paid in the male‐biased presumably competitive environments and that these survival effects occurred in the period after midwinter. Our study now points to one potential resource for which competition in the study of Nicolaus et al. (2012) occurred: the roosting box. In winter, especially males make use of roosting boxes (e.g., Krištín, Mihál, & Urban, 2001), which is also shown in our study by a higher probability to detect males roosting at midwinter. It could thus be that in the study of Nicolaus especially in the male‐biased environment at midwinter, competition for roosting boxes was high, leading to a survival cost of reproduction through a similar unknown mechanism as in our study.

Similar to our current study (Figure 3), Nicolaus et al. (2012) found that in noncompetitive environments (with a female‐biased sex ratio), family size manipulation seemed to have a positive effect on parental local survival. In the study of Nicolaus et al. (2012), this positive survival effect was hypothesized to be the consequence of (1) a relaxed overall level of competition in the local female‐biased environment due to an increased tendency of female fledglings to disperse and (2) a lower parental effort during postfledging care for the enlarged broods also due to a higher tendency to disperse of the female fledglings especially from enlarged broods because condition of these females was lower. In our study, such an explanation does not hold because sex ratio was not manipulated in the local environment, and this suggests that other effects may be at play.

#### 4.2.3 Alternative mechanism to explain a survival cost of reproduction under competition

We expected that parents would pay a survival cost of reproduction in our study due to an increased proportion of parents that raised larger experimental broods having to roost outside (e.g., higher thermoregulatory costs and/or higher predation risk; Drent, 1987; Mainwaring, 2011). In contrast, we found that experimental family size did not affect the ability of parents to claim a roosting box, but that parents in plots in which competition for roosting boxes was induced did pay a survival cost of reproduction. Our results show that survival cost of reproduction tended to be paid by those parents that claimed a roosting box after competition was induced (Figure 4). Potentially, depending on their experimental family size, parents survived differentially as a consequence of having to defend a roosting box during the period competition was induced. Survival costs associated with defending a box could occur in the following ways: (1) directly through injuries caused by fights or (2) through physiological/behavioral trade‐offs as a result of an increased defense needed to claim a roosting box (e.g., depletion of energy reserves, changes in endocrine status, and increased predation risk; e.g., Briffa & Sneddon, 2007; Dufty, 1989; Marler & Moore, 1988). Parents that raised larger experimental broods may have suffered more injuries due to fights or the effort needed to defend their roosting box may have gone at a greater expense of their perhaps already lower energy reserves/physiological status (see Appendix S1; Drent & Daan, 1980; Verhulst & Tinbergen, 1997; Sanz & Tinbergen, 1999; Tinbergen & Verhulst, 2000; Nilsson, 2002; Nicolaus et al., 2012; de Jong et al., 2014). In turn, this could have led to the observed survival cost of reproduction.

The pattern that parents that raised larger experimental broods that did claim a box had almost the same value of local survival as parents of the same manipulation group that roosted outside raises the question why these parents would compete for roosting boxes in the first place (Figure 4). One reason for this may be that roosting boxes were only removed for a short while. Perhaps the negative consequences of roosting outside on parental local survival would have been more severe if the experiment had lasted longer. The fitness payoff for parents to compete for a roosting box may in such a situation have been greater.

## 5 Conclusions and Implications

In contrast to our expectation, the occurrence of a survival cost of reproduction when competing for roosting boxes in winter could not be linked to a lower ability of parents to claim such a roosting box. The exact causal explanation why costs of reproduction are paid under increased competition thus remains unknown. Yet, the results of our study strengthen the claim that the occurrence of a survival cost of reproduction depends on the level of competition in the parents’ local environment (Nicolaus et al., 2012). Under high levels of competition, family size decisions can carry over to affect the future fitness of parents. This provides a potential causal explanation for the occurrence of density‐dependent effects on reproductive rates within populations (e.g., avian: Kluijver, 1951; Perrins, 1965; Both et al., 2000; Nicolaus et al., 2013; mammals: Morris, 1989; Koskela et al., 1999; Bonenfant et al., 2009). Under high population density, competition could exert a selective pressure on the family size decisions of parents and thus the reproductive rates in a population. This could occur through parents adaptively lowering their family size in the face of increased competition (Nicolaus et al., 2013: reaction norms) or by selection favoring those parents with a lower investment into current reproduction. This gives insight into how competition through selection on individual reproductive behavior could regulate population numbers.

## Conflict of Interest

None declared.
