# Supplementary material for: Is parental competitive ability in winter negatively affected by previous springs’ family size?
Source: Ecol Evol. 2017 Feb 3;7(5):1410–20. doi: 10.1002/ece3.2752 (PMC5330910; doi:10.1002/ece3.2752)
Supplement: Supplementary file 1 [file ECE3-7-1410-s001.docx]

## Appendix S1: effects of family size manipulation on parental feeding effort and the probability to produce a late brood

## Methods

### Measuring parental feeding effort

Both in 2012 and 2013, we tested whether parents increased their feeding effort in response to the family size manipulation (hereafter called ‘FS manipulation’; see methods accompanying study). To this end, in 2013, the colour ring scheme was altered and all caught parents that raised a manipulated brood, received an RFID transponder ring (catching occurred when the nestlings were 7 days old, see accompanying study; transponder type: EM4102 bird PIT tag 2.6mm, manufactured by: IB technology, Eccel Technology Limited). These transponder rings enabled us to measure the effect of FS manipulation on the number of feeding visits made by each of the parents to the nest when the nestlings were 12 days old (age around which brood energy demand peaks: van Balen 1973; Tinbergen and Dietz 1994; Sanz and Tinbergen 1999). The procedure of measuring the number of feeding visits was as follows: at the nestling age of 10 days, we fitted a dummy antenna around the flight hole in the box to get the birds acquainted to this novel object. At day 11 we replaced the dummy antenna with a real antenna fitted to a data logger (type: LID665, version V804, manufactured by Dorset identification b.v.). We used the number of feeding visits made by each of the parents during the whole following day (day 12) for subsequent analyses (00:00h to 00:00h; calculated using the same protocol as in electronic appendix S1 of Nicolaus et al. (2012)).

Next to the number of feeding visits per day, we measured the effect of FS manipulation on two other indexes of parental feeding effort, the change in brood weight after FS manipulation and the number of fledglings produced. The change in brood weight was determined by during both study years, at day 6, after family size manipulation weighing all nestlings individually (mass ± 0.1 g). When the nestlings were 14 days old we individually weighed all nestlings again to determine the change in brood weight since day 6. In those cases where all nestlings had died before day 14, the change in brood weight could not be calculated and we excluded these broods from subsequent analyses (N=13 broods). We additionally quantified whether FS manipulation increased the number of fledglings produced. These numbers were derived from fledge checks of all nest boxes 21 days after hatching. We analysed the data gathered on the number of fledglings produced twice, including and excluding all broods where parents failed to produce any fledglings (N=21 broods).

### Measuring the probability to start a late brood within the same breeding season

Using our standard protocol during the breeding season, late broods could be determined (see section in accompanying study: ‘monitoring egg laying and breeding’). Breeding females were either identified while sitting tight on eggs or when caught at the nest when the nestlings were 7 days old (see: accompanying study). From this we could calculate the probability of females (N=164) to produce a late brood within the same season (N=34; defined here as either a repeat brood: after an unsuccessful first brood or a second brood: after a successful first brood). We tested the effect of FS manipulation on the probability of females to start a late brood including and excluding the repeat broods, to check how the repeat broods (N= 5) affected our results. We did not focus on males because it more often occurred that they could not be caught at late broods (own population data).

### Statistics

We analysed the effects of FS manipulation on the change in brood weight using a linear mixed effects models (LMER) with a Gaussian error structure. The effect of family size manipulation on the number of visits made by each parent to the nest and the number of fledglings produced were done using a generalized linear mixed effects model (GLMER) with a Poisson error structure. The effect of FS manipulation on the probability of parents to start a late brood was analysed using generalized linear mixed effects models (GLMER) with a Binomial error structure.

Within all analyses, FS manipulation was included as a continuous variable because of our directional expectation (directional statistical tests, see: Knowles et al. 2009, Fokkema et al. 2016) and we allowed for non-linear effects by including a quadratic effect of FS manipulation. Next to this, we included three other predictor variables: 1) study year, this factor was included in all analyses except the analysis of the number of visits/day made by each parent, as we only had one year of measurements, 2) sex of the parent, this factor was included in the analyses of the number of visits per day made by each parent. Sex of the parent was not included in the other analyses as these were done at the brood level. We included two random variables: 1) ‘trio’ number, this factor was included as a random effect in all analyses to correct for non-independence of the matched trios of nests (see methods accompanying study: ‘family size manipulation’), 2) brood ID, this identification number for the brood raised, was included as a factor in our analyses of the number of visits/day by each parent to account for the non-independence within breeding pairs. Model selection was done using a backwards elimination procedure similar as reported in the accompanying study.

## Results:

### Family size manipulation and parental feeding effort

Two of three measured indexes of parental effort increased with FS manipulation. Between years the relationship between FS manipulation and the number of fledglings produced differed (table S1; FS manipulation^2^ x year 2013: intercept: 1.94 ± 0.09, β: 0.05 ± 0.02, χ^2^_d.f.1_ = 9.38, P < 0.01, FS manipulation x year 2013: χ^2^_d.f.1_= 0.53, P = 0.47). But, on the whole, FS manipulation had a positive effect of the number of fledglings produced (FS manipulation: β = 0.09 ± 0.01, χ^2^_d.f.1_= 40.79, P < 0.001). When the analysis of the effect of FS manipulation on the number of fledglings produced was run excluding broods in which no nestlings fledged, the difference between years in the quadratic effect of family size manipulation disappeared (FS manipulation^2^ x year 2013: intercept: 2.01 ± 0.07, β: 0.02 ± 0.02, χ^2^_d.f.1_ = 1.99, P = 0.15) . All other effects were similar.

The number of feeding visits per day of both parents in 2013 also increased with FS manipulation, but the effect differed between the sexes. For males the effect of FS manipulation on the number of feeding visits was stronger than for females and did not level off at the enlarged broods (FS manipulation x sex: males: intercept: 5.67 ± 0.11, β = 0.03 ± 0.006, χ^2^_d.f.1_= 26.25, P <0.001; FS manipulation^2^ x sex: males: β: 0.05 ± 0.003, χ^2^_d.f.1_ = 185.88, P < 0.001).

The change in brood weight between day 6 and day 14 days was positively but not significantly affected by FS manipulation (FS manipulation: intercept: 52.23 ± 3.39, β = 1.16 ± 0.85, χ^2^_d.f.1_ = 1.86, P = 0.17).

### Family size manipulation and the probability of females to start a late brood

In 2013, but not in 2012, FS manipulation negatively affected the probability of females to start a late brood within the same breeding season (repeat- and second broods; FS manipulation x year 2013: intercept: -1.75 ± 0.30, β = -0.39 ± 0.17, χ^2^_d.f.1_ = 4.98, P < 0.05). There was no evidence for a non-linear effect of FS manipulation on the probability to produce a late brood (FS manipulation^2^: χ^2^_d.f.1_ = 0.55, P = 0.35; FS manipulation^2^ x year 2013: χ^2^_d.f.1_= 1.40, P = 0.24). When the repeat broods were excluded from the analysis (e.g. only parents with successful first broods included) effects were similar.

## References

van Balen JH. 1973. A Comparative Study of the Breeding Ecology of the Great Tit Parus Major in Different Habitats. Ardea 61:1–93.

Fokkema RW, Ubels R, Tinbergen JM. 2016. Great tits trade off future competitive advantage for current reproduction. Behav. Ecol. 27:1656–1664.

Knowles SCL, Nakagawa S, Sheldon BC. 2009. Elevated reproductive effort increases blood parasitaemia and decreases immune function in birds: A meta-regression approach. Funct. Ecol. 23:405–415.

Sanz JJ, Tinbergen JM. 1999. Energy expenditure, nestling age, and brood size: an experimental study of parental behavior in the great tit Parus major. Behav. Ecol. 10:598–606.

Tinbergen JM, Dietz M. 1994. Parental energy expenditure during brood rearing in the Great Tit (Parus major) in relation to body mass, temperature, food availability and clutch size. Funct. Ecol. 8:563–572.

## Tables

**Table S1:** Overview of the effect of family size manipulation on the number of feeding visits made by each parent (male and female) in 2013 to the nest and for 2012 and 2013 on the weight change of the whole brood (brood mass measured at the nestling age of 14 days minus the brood mass measured right after manipulation at the nestling age of 6 days) and the final number of fledglings produced. Averages per manipulation group are depicted.

| # of nestlings  exchanged | # visits per day  (SE\|N)  Male Female | | Change in brood weight in grams  (SE\|N)  2012 2013 | | # of fledglings  (SE\|N)  2012 2013 | |
| --- | --- | --- | --- | --- | --- | --- |
| -2 | 184 (8\|2) | 136 (42\|2) | 40.8 (3.4\|3) | 25.5 (12.3\|4) | 4.3 (0.3\|3) | 3.8 (0.6\|4) |
| 0 | 147 (51\|2) | 300 (118\|2) | 55.6 (4.7\|3) | 37.3 (15.9\|3) | 6.3 (0.3\|3) | 4.0 (0.6\|4) |
| 2 | 172 (14\|2) | 372 (53\|2) | 57.1 (9.8\|3) | 10.6 (10.2\|4) | 7.7 (0.3\|3) | 6.3 (0.9\|4) |
| -3 | 247 (31\|10) | 227 (31\|10) | 46.1 (2.9\|26) | 39.8 (2.9\|20) | 4.6 (0.4\|28) | 4.7 (0.4\|21) |
| 0 | 298 (62\|8) | 314 (49\|8) | 59.8 (2.8\|26) | 43.8 (8.8\|17) | 7.3 (0.5\|28) | 4.5 (0.8\|21) |
| 3 | 438 (73\|9) | 293 (45\|9) | 52.2 (7.3\|27) | 49.6 (9.4\|19) | 8.0 (0.8\|28) | 7.2 (1.0\|21) |
